# Shortest pulmonary vein atrial fibrillation cycle length identifies pulmonary vein isolation responders beyond clinical atrial fibrillation pattern: the FARS-AF II study

**DOI:** 10.1093/europace/euag033

**Published:** 2026-02-23

**Authors:** Lorenzo Marcon, Marco Bergonti, Francesco Spera, Johan Saenen, Wim Huybrechts, Hielko Miljoen, Olivier Van Leuven, Lien Vandaele, Anouk Wittock, Hein Heidbuchel, Andrea Sarkozy

**Affiliations:** Department of Cardiology, Antwerp University Hospital, Wilrijkstraat 10, Edegem, Antwerp 2650, Belgium; Heart Rhythm Management Centre, Universitair Ziekenhuis Brussel, Heart Rhythm Research Brussels, Postgraduate Program in Cardiac Electrophysiology and Pacing, Vrije Universiteit Brussel, European Reference Networks Guard-Heart, Avenue du Laerbeek 101, Jette, Brussels 1090, Belgium; Department of Cardiology, Antwerp University Hospital, Wilrijkstraat 10, Edegem, Antwerp 2650, Belgium; Division of Cardiology, Cardiocentro Ticino Institute, Ente Ospedaliero Cantonale, Lugano, Switzerland; Department of Cardiology, Antwerp University Hospital, Wilrijkstraat 10, Edegem, Antwerp 2650, Belgium; Cardiovascular Department, Sant’ Andrea University Hospital, Rome, Italy; Department of Cardiology, Antwerp University Hospital, Wilrijkstraat 10, Edegem, Antwerp 2650, Belgium; Department of Cardiology, Antwerp University Hospital, Wilrijkstraat 10, Edegem, Antwerp 2650, Belgium; Department of Cardiology, Antwerp University Hospital, Wilrijkstraat 10, Edegem, Antwerp 2650, Belgium; Research Group Cardiovascular Diseases, Department GENCOR, University of Antwerp, Prinsstraat 13, Antwerp 2000, Belgium; Department of Cardiology, Antwerp University Hospital, Wilrijkstraat 10, Edegem, Antwerp 2650, Belgium; Department of Cardiology, Antwerp University Hospital, Wilrijkstraat 10, Edegem, Antwerp 2650, Belgium; Anesthesiology Department, University Hospital Antwerp, Antwerp, Belgium; Department of Cardiology, Antwerp University Hospital, Wilrijkstraat 10, Edegem, Antwerp 2650, Belgium; Research Group Cardiovascular Diseases, Department GENCOR, University of Antwerp, Prinsstraat 13, Antwerp 2000, Belgium; Department of Cardiology, Antwerp University Hospital, Wilrijkstraat 10, Edegem, Antwerp 2650, Belgium; Heart Rhythm Management Centre, Universitair Ziekenhuis Brussel, Heart Rhythm Research Brussels, Postgraduate Program in Cardiac Electrophysiology and Pacing, Vrije Universiteit Brussel, European Reference Networks Guard-Heart, Avenue du Laerbeek 101, Jette, Brussels 1090, Belgium; Research Group Cardiovascular Diseases, Department GENCOR, University of Antwerp, Prinsstraat 13, Antwerp 2000, Belgium

**Keywords:** Atrial fibrillation, Atrial fibrillation physiopathology, Pulmonary vein isolation, Catheter ablation, Pulmonary vein activity, Arrhythmia recurrence

## Abstract

**Aims:**

Atrial fibrillation cycle length (AF-CL) measured in the pulmonary veins (PVs) with a novel simple method [the average of the 10 consecutive Fastest Atrial Repetitive Similar signal interval (FARS_10_)] accurately identified pulmonary vein isolation (PVI) responders in a preliminary study. This study aims to evaluate differences in PV-FARS_10_ between paroxysmal and persistent AF and to define the optimal cut-off to predict PVI-only approach success in a large population.

**Methods and results:**

We prospectively enrolled consecutive patients with persistent or paroxysmal AF undergoing first PVI in a single-centre study. The primary endpoint was atrial arrhythmia recurrence. A total of 219 patients (61.8 ± 11.2 years, 25.1% female) were included, with 70 patients (32%) having paroxysmal AF and 149 patients (68%) persistent AF. After a median follow-up of 18.0 [interquartile range (IQR) 10.2–42.3] months, 72 (32.9%) patients experienced AF/atrial flutter (AFL)/atrial tachycardia (AT) recurrence. Patients with shortest PV-FARS_10_ ≤ 155 ms had a lower rate of AF/AFL/AT recurrence compared to those with shortest PV-FARS_10_ > 155 ms in the overall population (HR 0.34, *P* < 0.001), in persistent AF (HR 0.40, *P* = 0.002), and in paroxysmal AF (HR 0.18, *P* = 0.01). In multivariable analysis—which included age, sex, body mass index, CHA_2_DS_2_-VA score, obstructive sleep apnoea syndrome, duration of AF, AF type (paroxysmal vs. persistent), left ventricular ejection fraction, left atrial volume index, shortest PV-FARS_10_/left atrial appendage-FARS_10_, and AF termination during ablation—only the shortest PV-FARS_10_ ≤ 155 ms was the significant predictor of AF/AFL/AT recurrence-free survival in the overall population (HR 0.45, CI: 0.26–0.78, *P* = 0.005). Paroxysmal AF patients more frequently had shortest PV-FARS_10_ ≤ 155 ms than persistent AF patients (61.4% vs. 42.3%, *P* = 0.009).

**Conclusion:**

PV-FARS_10_ can accurately identify PVI responders among patients with persistent and paroxysmal AF. Patients with slow PV (shortest PV-FARS_10_ > 155 ms) experience a higher rate of AF/AFL/AT recurrence after PVI-only approach. The shortest PV-FARS_10_ ≤ 155 ms occurs more frequently in paroxysmal AF patients than in persistent AF patients.

## Graphical Abstract

**Graphical Abstract:**
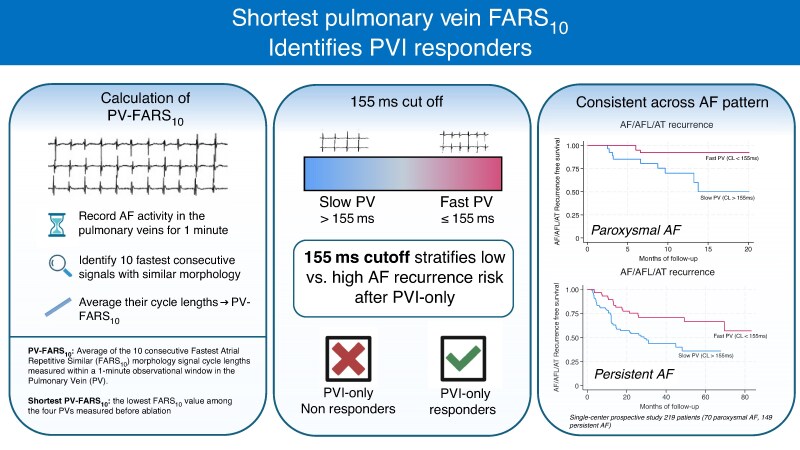
Left: Calculation of the pulmonary vein (PV) FARS_10_ PV activity is recorded for 1 min and the 10 fastest consecutive atrial repetitive similar signals are averaged. Center: A cut-off of 155 ms stratifies patients with ‘fast PV’ (≤155 ms, lower recurrence risk after PVI-only, i.e. PVI-only responders) and ‘slow PV’ (>155 ms, higher recurrence risk, i.e. PVI-only non-responders). Right: The predictive value of the 155 ms cut-off was consistent in both paroxysmal and persistent AF patients.

What’s new?

- A novel and simple method (FARS_10_) for measuring atrial fibrillation (AF) cycle length (CL) in the pulmonary veins (PV) accurately identifies pulmonary vein isolation (PVI) responders.
- A cut-off value of 155 ms stratifies patients with ‘fast PV’ (≤155 ms, lower recurrence risk after PVI-only, i.e. PVI responders) and ‘slow PV’ (>155 ms, higher recurrence risk, i.e. PVI non-responders).
- The predictive value of the 155 ms cut-off is consistent across both paroxysmal and persistent AF patients.

## Introduction

**Figure 1:**
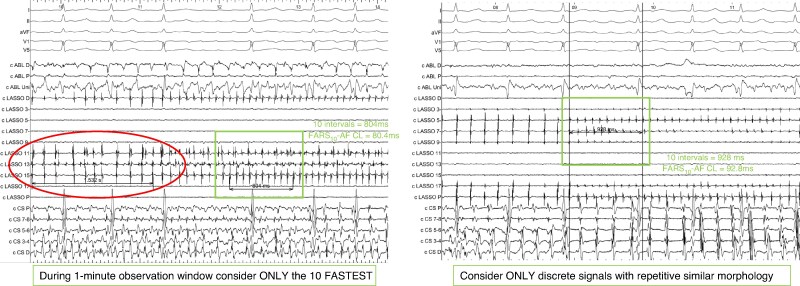
How to measure FARS_10_. The measurements shown within the square box are correct. During a 1-min observation window, the duration of the fastest consecutive 10 intervals is measured and divided by 10 to calculate the mean FARS_10_. Fragmented signals, signals with changing morphology, double potentials, or transient declaration (illustrated within the oval) should be avoided in the measurements.

Durable pulmonary vein isolation (PVI) has up to 80% 1-year success rate in paroxysmal atrial fibrillation (AF); however, the success rate of PVI-only approach in persistent AF is lower, approaching 70% with new pulsed field ablation technologies.^1–6^ Attempts to broaden the ablation lesion set beyond PVI have not resulted in consistent improvements in outcomes.^7,8^ A better identification of the up to two-thirds of patients responding to PVI-only approach in persistent AF would facilitate the development and testing of novel patient-tailored additional ablation strategies. Testing novel ablation strategies only in PVI non-responders would allow to document higher treatment effect in case of effective PVI-Plus strategies. Additionally, extensive and potentially proarrhythmic unnecessary ablation lesions could be avoided in PVI responders. Moreover, clinical parameters alone were suboptimal to predict the finding of durable PVI at redo procedures in case of recurrent AF.^9,10^ Accordingly, real-world practice shows substantial heterogeneity in contemporary strategies for repeat AF ablation, and the optimal approach remains to be defined.^11^ Our group developed a novel method to identify PVI responders by characterizing PV refractory periods by examining PV AF cycle lengths (CLs). Additionally, our method reproducibly identified only transient fast repetitive electrical activity in the pulmonary veins (PVs). The exclusions of continuous or changing fragmented signals and double potentials from the measurements assure that the measured AF-CL correctly mirrors tissue refractory periods. We reported in a preliminary study that the shortest PV AF-CL measured with this novel simple method [the average of the 10 consecutive Fastest Atrial Repetitive Similar morphology signal cycle lengths (FARS_10_) in a 1-min observational window—*Figure 1*] accurately identified PVI responders among patients with persistent AF.^12^ Another prospective multicentre study by our group (the INDUCE-AF trial) confirmed the predictive value of shortest PV-FARS_10_ also in patients with paroxysmal AF.^13^ These findings support the hypothesis that transiently fast regularized regions could be triggers of paroxysmal AF or drivers of persistent AF or are located in close proximity to these regions. The aims of the present study were (i) to conduct a comprehensive assessment of the predictive value of PV-FARS_10_ in a patient population of both paroxysmal and persistent AF and (ii) to evaluate differences in PV-FARS_10_ between paroxysmal and persistent AF patients.

## Methods

### Study population

Patients with paroxysmal and persistent AF undergoing first AF ablation were enrolled in our prospective study at the Antwerp University Hospital. This study included a subset of 100 patients with persistent AF and 70 patients with paroxysmal AF reported in previous studies.^12,13^ All consecutive patients undergoing first ablation with PVI-only approach between 2015 and 2022 were screened for this study. Patients presenting in AF or induced to AF prior to mapping and with PV-FARS_10_ measured in all PVs were included. Exclusion criteria were non-inducibility of sustained AF, additional ablation beyond PVI (except cavotricuspid isthmus ablation), or participation in a competing research study. Detailed inclusion and exclusion criteria are provided in the Supplementary methods. The study was carried out according to the principles of the Declaration of Helsinki. All participants provided informed consent for inclusion in the study, and the study was approved by the ethical committee of the Antwerp University Hospital.

### Study description

Atrial fibrillation cycle length was measured using the FARS_10_ method with a decapolar catheter positioned in the superior vena cava (SVC), right atrial appendage (RAA), and coronary sinus (CS) and a circular mapping catheter (Lasso or Achieve) positioned in the left atrial appendage (LAA) and PVs. Shortest PV-FARS_10_ was defined as the lowest FARS_10_ value among the four pulmonary veins measured before ablation. Further details on FARS_10_ acquisition and measurement are provided in the Supplementary material. Briefly, FARS_10_ measurements were performed according to a standardized protocol (fixed 1-min window; average of fastest 10 consecutive repetitive similar morphology intervals; avoidance of continuously fragmented signals and double potentials), and PV recordings were acquired before any ablation. Reproducibility of FARS_10_ measurements has been previously reported^12^; the inter-/intra-observer variability was not reassessed in the present cohort. High-density bipolar voltage mapping of LA was performed in 101 patients with persistent AF. Presence of low-voltage zone (LVZ) was defined as >5% LA surface area of abnormal bipolar voltage during high-density bipolar voltage based on our MASH-AF II study.^14^ High-burden LVZ was defined as >15% LA surface area, in accordance with the MASH-AF II study identifying this threshold as prognostically relevant in persistent AF.^12^ The abnormal bipolar voltage cut-off was set at ≤0.5 mV during sinus rhythm and ≤0.24 mV during AF based on our previous validation work.^15^ Patients were evaluated at 3, 6, and 12 months in the first year and then every 6–12 months or if symptoms developed. At each visit, ambulatory 24–48 h Holter monitoring was performed.

### Endpoint

The primary endpoint was the absence of documented recurrence of AF or atrial tachycardia (AT) or atrial flutter (AFL) lasting for >30 s after the initial 3-month blanking period. The shortest PV-FARS_10_ activity was evaluated in relation to the primary endpoint, and the correlation between PV activity and other baseline variables was assessed.

### Ablation procedure

The discontinuation of anti-arrhythmic drugs (AADs) prior to the procedure was left to the operators’ discretion (see Supplementary methods). In general, AADs were continued and withhold only on the day of the procedure. The catheter ablation technique used for PVI by means of radiofrequency energy (RF) or Cryoballoon (Cryo) is described in detail in the Supplementary material. Briefly, all patients underwent either point-by-point RF ablation guided by automatic lesion annotation with minimum force–time integral and, later, ablation index targets using a 3D mapping system, or second-generation Cryo ablation.

### Statistical analysis

**Figure 2:**
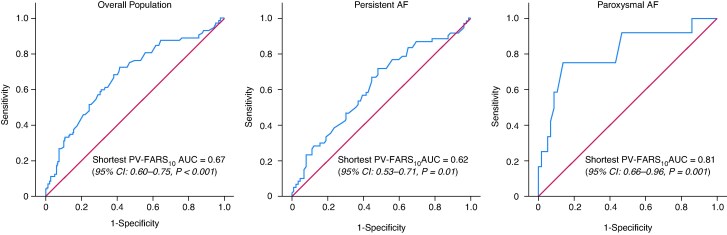
ROC curve analysis for the prediction of PVI responders based on shortest PV-FARS_10_, in the overall study population and in subgroups of patients with paroxysmal and persistent AF. In the first graph (overall population, *n* = 219), a cut-off of 155 ms is associated with a sensitivity of 72% and a specificity of 58%. In the middle graph (persistent AF, *n* = 149), the same cut-off yields a sensitivity of 72% and a specificity of 52%. In the last graph (paroxysmal AF, *n* = 70), a cut-off of 155 ms is associated with a sensitivity of 75% and a specificity of 69%.

Continuous variables were expressed as mean ± standard deviation or median and interquartile range as appropriate and categorical values as frequencies (percentages). Comparisons between groups were undertaken with parametric (Student’s *t*-test) or non-parametric (Mann–Whitney *U* test) test when appropriate. The χ^2^ or Fisher’s exact test was used to compare categorical variables. Kaplan–Meier survival analysis was performed to analyse the cumulative event rates, and the log-rank test was used to detect significant differences between groups. Cox proportional hazard regression analysis was used to assess the relationship between baseline characteristics and AF/AT/AFL recurrence during follow-up. Only variables with *P* < 0.05 in univariable analysis were used for the multivariable analysis. ROC curve was used to assess the predictive value of PV-FARS_10_ activity and to determine the optimal cut-off for distinguishing fast and slow shortest PV-FARS_10_, for both the overall population and patients with persistent and paroxysmal AF (*Figure 2*; *Table 1*). A *P* value of <0.05 was considered to be statistically significant. The SPSS Statistics 29.0.2.0 (IBM Corp, Armonk, New York, USA) was used for all statistical analyses.

**Table 1:** ROC curve analysis for prediction of PVI responders according to shortest PV-FARS_10_ cut-off value of 155 ms in the total study population and in the subgroup of patients with paroxysmal and persistent AF

Shortest PV-FARS_10_	Area under the curve (Area, asymptotic significance, asymptotic 95% confidence interval)	Cut-off (ms)	Sensitivity, specificity
**Total population *n* = 219**	0.67, <0.001, 0.60–0.75	155	72%, 58%
**Persistent AF *n* = 149**	0.62, 0.01, 0.53–0.71	155	72%, 52%
**Paroxysmal AF *n* = 70**	0.81, 0.001, 0.66–0.96	155	75%, 69%

Continuous variables are shown as mean ± SD or median and IQR. Discrete variables are presented as numbers and percentages (%).

PV, pulmonary vein; CL, cycle length; FARS_10_, 10 consecutive Fastest Atrial Repetitive Similar morphology signal; AF, atrial fibrillation.

## Results

### Patient population and baseline characteristics

The study population consisted of 219 patients (61.8 ± 11.2 years, 25.1% female) undergoing first AF ablation, with 70 patients (32%) having paroxysmal and 149 patients (68%) persistent AF. *Table 2* reports the baseline characteristics stratified according to the clinical type of AF, while Supplementary material online, *Table S1*, reports the baseline characteristics stratified according to the shortest PV-FARS_10_ activity. Compared to patients with paroxysmal AF, those with persistent AF had higher BMI, more dilated LA, higher prevalence of previous coronary artery disease and obstructive sleep apnoea syndrome (OSAS), and slower PV-FARS_10_ activity. Patients with fast PV-FARS_10_ were younger, were more frequently male, had a shorter duration of AF history, and had more frequently paroxysmal AF. *Table 3* displays the percentage of LVZ categorized based on the PV activity, revealing a statistically significant lower rate of LVZ among patients with fast PV-FARS_10_. Furthermore, correlation analysis demonstrated a significant association between higher PV-FARS_10_ values and increased prevalence of LVZ (HR 1.20, 95% CI: 1.06–1.35, *P* = 0.003). Baseline data stratified according to AF/AFL/AT recurrence during follow-up are reported in Supplementary material online, *Table S2*.

**Table 2:** Baseline characteristics of the study population stratified by clinical AF pattern

Characteristic	Total *N* = 219	Persistent AF *N* = 149	Paroxysmal AF *N* = 70	*P* value
**Age, years**	61.8 ± 11.2	62.4 ± 9.4	60.6 ± 14.3	0.27
**Female sex**	55 (25.1%)	37 (24.8%)	18 (25.7%)	0.87
**Hypertension**	111 (50.7%)	78 (52.3%)	33 (47.1%)	0.56
**Diabetes**	28 (12.8%)	23 (15.4%)	5 (7.1%)	0.12
**BMI, kg/m^2^**	28.5 ± 4.93	29.4 ± 5.2	26.4 ± 3.5	<0.001
**Coronary artery disease**	34 (15.5%)	29 (19.5%)	5 (7.1%)	0.02
**CHA_2_DS_2_-VA score**	2 (0–3)	2 (1–3)	1 (0–2)	0.004
**Obstructive sleep apnoea**	33 (15.1%)	28 (18.8%)	5 (7.1%)	0.02
**LVEF, %**	56.9 ± 9.3	55.5 ± 10.9	59.9 ± 2.4	0.001
**LAVI, mL/m^2^**	39.5 ± 12.4	42.0 ± 11.3	33.5 ± 12.9	<0.001
**Years since the first AF episode**	2.1 (0.8–5.3)	1.8 (0.7–5.2)	2.5 (1.1–5.5)	0.39
**Patients on flecainide**	51 (23.3%)	31 (20.8%)	20 (28.6%)	0.23
**Patients on sotalol**	54 (24.7%)	38 (25.5%)	16 (22.9%)	0.74
**Patients on amiodarone**	42 (19.2%)	36 (24.2%)	6 (8.6%)	0.006
**Procedure-related information**				
**Fast PV ≤ 155 ms**	106 (48.4%)	63 (42.3%)	43 (61.4%)	0.009
**AF termination during procedure**	71 (32.6%)	37 (25%)	34 (48.6%)	<0.001
**Cryoballon ablation—no. (%)**	52 (23.8%)	0 (0%)	52 (74.3%)	–
**Radiofrequency ablation—no. (%)**	167 (76.2%)	149 (100%)	18 (25.7%)	<0.001

Continuous variables are shown as mean ± SD or median and IQR. Discrete variables are presented as numbers and percentages (%).

PV, pulmonary vein; BMI, body mass index; LVEF, left ventricular ejection fraction; LAVI, left atrial volume index; FARS_10_, 10 consecutive Fastest Atrial Repetitive Similar morphology signal.

**Table 3:** Correlation between PV-FARS_10_, clinical outcome, and LVZs in patients with persistent AF (*n* = 149)

CHARACTERISTICS	TOTAL *N* = 149	FAST PV (≤155 MS) *N* = 63	SLOW PV (>155 MS) *N* = 86	*P* VALUE
**LVZ**	37/101 (36.6%)	10/44 (22.7%)	27/57 (47.4%)	0.01
**High burden LVZ (>15% LA area)**	29/101 (28.7%)	7/44 (15.9%)	22/57 (38.6%)	0.01

Characteristics	Total *n* = 149	AF/AFL/AT recurrence *n* = 60	AF/AFL/AT no recurrence *n* = 89	*P* value
**LVZ**	37/101 (36.6%)	16/39 (41.0%)	21/62 (33.9%)	0.52
**High burden LVZ (>15% LA area)**	29/101 (28.7%)	13/39 (33.3%)	16/62 (25.8%)	0.52

Continuous variables are shown as mean ± SD or median and IQR. Discrete variables are presented as numbers and percentages (%).

AF, atrial fibrillation; AFL, atrial flutter; AT, atrial tachycardia; LVZ, low-voltage zone; LA, left atrium.

### Procedural data

All patients underwent successful PVI with RF (76.2%) or Cryo (23.8%) ablation (*Table 2*). Specifically, Cryo was performed in 52 (74.3%) paroxysmal AF patients, whereas the remaining 18 (25.7%) underwent RF ablation. All patients in the persistent AF group underwent RF ablation. Atrial fibrillation termination during procedure was documented in 32.6% of patients. Atrial fibrillation termination was more frequent in paroxysmal AF (48.6%) than in persistent AF (25%) and in patients with fast PV (40%) as compared to slow PV (25.7%). These data are presented in *Table 2* and Supplementary material online, *Table S1*. Supplementary material online, *Table S3*, presents FARS_10_-AF-CL in the PVs, CS, LAA, RAA, and SVC based on the clinical type of AF. There was no statistically significant difference in shortest PV-FARS_10_ between patients with paroxysmal and persistent AF, as well as in FARS_10_ of other atrial structures. The distributions of shortest PV-FARS_10_ in paroxysmal and persistent AF patients are shown in Supplementary material online, *Figure S1*. Supplementary material online, *Table S4*, reports FARS_10_ measurements across various atrial structures between patients with fast and slow PV. The FARS_10_ stratified according to arrhythmic recurrence during follow-up is reported in Supplementary material online, *Table S5*. Patients with AF/AFL/AT recurrence are characterized by a statistically significant slower PV-FARS_10_, regardless of their clinical type of AF. The FARS_10_ stratified according to the AAD therapy is reported in Supplementary material online, *Table S7*. In patients under AAD therapy, all the PVs were slower as compared to patients not treated with these drugs. Eighty-five patients (38.8%) were in sinus rhythm at the start of the procedure and AF was induced; subgroup comparisons between induced and spontaneous AF are reported in Supplementary material online, *Table S8*. Induced AF was associated with shorter PV-FARS_10_ and higher rates of fast PV (<155 ms) and AF termination (see Supplementary material online, *Table S8*). Supplementary material online, *Table S9*, shows that AF termination during ablation predominantly occurred at the fastest PV, particularly when the shortest PV-FARS_10_ was ≤155 ms.

### Follow-up

**Figure 3:**
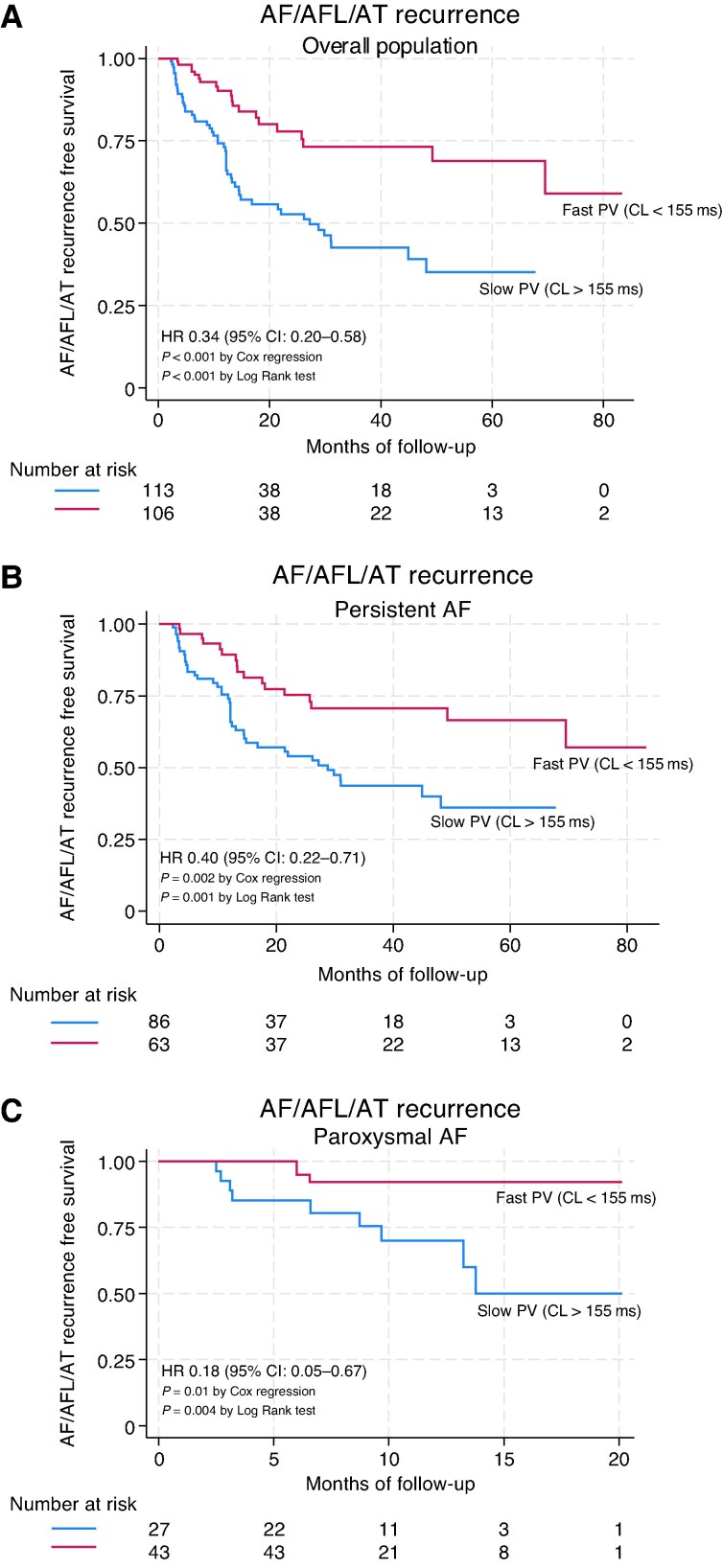
(*A*) AF/AFL/AT recurrence-free survival in overall population. (*B*) AF/AFL/AT recurrence-free survival in persistent AF patient. (*C*) AF/AFL/AT recurrence-free survival in paroxysmal AF patient according to pulmonary vein activity.

During a median follow-up period of 18.0 (10.2–42.3) months, 72 (32.9%) patients experienced AF/AFL/AT recurrence. The median follow-up duration for paroxysmal AF patients [11.8 (8.4–14.6) months] was shorter than that of persistent AF [34.1 (13.4–53.7) months]. Using ROC curve analysis, shortest PV-FARS_10_ was identified as a reliable predictor of PVI responders in both the overall population and in persistent and paroxysmal AF (*Figure 2*). A shortest PV-FARS_10_ cut-off of 155 ms was found to be the optimal for distinguishing patients with and without recurrence during follow-up, with a sensitivity of 72% and a specificity of 58% in the overall population (AUC 0.67, 95% CI: 0.60–0.75, *P* < 0.001). In persistent AF, the 155 ms cut-off value had a sensitivity of 72% and a specificity of 52% (AUC 0.62, 95% CI 0.53–0.71, *P* = 0.04) and in paroxysmal AF, a sensitivity of 75% and a specificity of 69% (AUC 0.81, 95% CI 0.66–0.96, *P* = 0.001) to identify PVI responders. These findings are presented in *Table 1* and *Figure 2*. A value of 155 ms was utilized to distinguish patients who had slow (>155 ms) and fast (≤155 ms) PV activity. Supplementary material online, *Table S6*, presents the follow-up data of patients based on the clinical type of AF, whereas *Table 4* presents the follow-up data of patients according to the PV-FARS_10_ activity. In the overall population, patients with slow PV-FARS_10_ experienced more than twice the recurrence rate compared to patients with fast PV-FARS_10_ (46% vs. 18.9%). Compared to patients with slow PV-FARS_10_, the rate of recurrence in patients with fast PV-FARS_10_ was 10% vs. 27% at 1 year, 22% vs. 47% at 2 years, and 27% vs. 61% at 4 years, log-rank *P* < 0.001 (*Figure 3A*), HR 0.34 (CI: 0.20–0.58), *P* < 0.001. In patients with persistent AF, the rate of recurrence was 11% vs. 26% at 1 year, 25% vs. 46% at 2 years, and 30% vs. 64% at 4 years, log-rank *P* = 0.001 (*Figure 3B*), HR 0.40 (CI: 0.22–0.71), *P* = 0.002. In patients with paroxysmal AF, the rate of recurrence was 8% vs. 25% at 1 year, log-rank *P* = 0.004 (*Figure 3C*), HR 0.18 (CI: 0.05–0.67), *P* = 0.01. In univariable analysis, left atrial volume index (LAVI), AF termination during ablation, and the shortest PV-FARS_10_ ≤ 155 ms were predictors of AF/AFL/AT recurrence-free survival in the overall population. The PV/LAA-FARS_10_ ratio was not a significant predictor of AF recurrence in the overall cohort, although a borderline association was noted (HR 3.5, *P* = 0.06, 95% CI: 0.95–13.0). In multivariable analysis, only the shortest PV-FARS_10_ ≤ 155 ms was the significant predictor of AF/AFL/AT recurrence-free survival in the overall population (*Table 5*). Supplementary material online, *Table S10*, presents redo procedures and PV reconnection rates.

**Table 4:** Comparison of clinical outcomes between patients with fast (≤155 ms) and slow (>155 ms) shortest PV-FARS_10_

CHARACTERISTIC	TOTAL *N* = 219	FAST PV (≤155 MS) *N* = 106	SLOW PV (>155 MS) *N* = 113	*P* VALUE
**AF/AFL/AT recurrence**	72 (32.9%)	20 (18.9%)	52 (46%)	<0.001
**AF recurrence**	27 (12.3%)	7 (6.6%)	20 (17.7%)	0.01
**AT/AFL recurrence**	45 (20.5%)	13 (12.3%)	32 (28.3%)	<0.01
**Redo procedure**	27 (12.3%)	10 (9.4%)	17 (15%)	0.22
**PV reconnection at redo**	9/27 (33.3%)	5/10 (50.0%)	4/17 (23.5%)	0.22
**AF/AFL/AT recurrence after redo procedure**	7/18 (38.9%)	1/7 (14.3%)	6/11 (54.5%)	0.15

Continuous variables are shown as mean ± SD or median and IQR. Discrete variables are presented as numbers and percentages (%).

AF, atrial fibrillation; AFL, atrial flutter; AT, atrial tachycardia.

**Table 5:** Predictors of AF/AFL/AT recurrence, overall population

	UNIVARIATE ANALYSIS				MULTIVARIATE ANALYSIS			
	HR	95% CI		*P* VALUE	HR	95% CI		*P* VALUE
		LOWER	UPPER			LOWER	UPPER	
**Female sex**	1.27	0.77	2.10	0.35				
**BMI**	0.99	0.95	1.04	0.82				
**Years of AF**	0.99	0.96	1.02	0.62				
**Age**	1.02	0.99	1.05	0.06				
**CHA_2_DS_2_-VA**	1.08	0.95	1.24	0.22				
**OSAS**	1.33	0.73	2.43	0.35				
**LVEF**	0.98	0.95	1.01	0.30				
**LAVI, mL/m^2^**	1.02	1.01	1.04	0.04	1.02	0.99	1.03	0.08
**AF termination during ablation**	0.55	0.31	0.99	0.04	0.58	0.31	1.10	0.09
**Shortest PV**-**FARS_10_ ≤ 155 ms**	0.36	0.21	0.60	< 0.001	0.45	0.26	0.78	0.005
**Shortest PV-FARS_10_/LAA-FARS_10_**	3.52	0.95	13.06	0.06				
**Paroxysmal AF**	1.26	0.88	1.80	0.20				
**Persistent AF**	1.33	0.70	2.55	0.38				

Univariate (column on the left) and multivariate (on the right) Cox regression analysis for predictors of AF recurrence after PVI.

AF, atrial fibrillation; BMI, body mass index; CL, cycle length; FARS_10_, 10 consecutive Fastest Atrial Repetitive Similar morphology signal; I, inferior; LAVI, left atrial volume index; LVEF, left ventricular ejection fraction; OSAS, obstructive sleep apnoea; PV, pulmonary vein; AFL, atrial flutter; AT, atrial tachycardia; LAA, left atrial appendage; RAA, right atrial appendage.

## Discussion

The main findings of our study are as follows: (i) the cut-off value of 155 ms for shortest PV-FARS_10_ identifies patients with AF/AFL/AT recurrence following PVI-only approach with reasonable sensitivity, although moderate specificity in both the overall population and patients with persistent and paroxysmal AF (*Graphical Abstract*). Patients with slow PVs (shortest PV-FARS_10_ > 155 ms) exhibit more than twice higher rate of recurrence compared to those with fast PVs (shortest PV-FARS_10_ ≤ 155 ms). (ii) There is no significant difference in shortest PV-FARS_10_ between persistent and paroxysmal AF patients; however, paroxysmal AF patients more frequently have the shortest PV-FARS_10_ ≤ 155 ms.

### Cycle length measurement in atrial fibrillation

More than 20 years ago, the measurement of AF-CL was proposed as a method to characterize PV trigger activity in paroxysmal AF and to identify the drivers maintaining AF in persistent AF.^16–19^ Obtaining a reproducible and accurate measurement of AF-CL mirroring tissue refractoriness was challenging due to the distinctive electrogram characteristics of fibrillatory conduction. The primary obstacle to precise AF-CL measurement is the dynamic changes in signal morphology, including double potentials and fragmented and non-fragmented signals with or without considering an arbitrary minimum refractory period or signal duration. These complex signals may be a result of the summation of local activation wavefronts or may include far-field signals. Additionally, an average AF-CL measurement over a fixed longer period (e.g. 1 min) may miss transient firing. Specifically, when AF-CL is computed as the mean of all consecutive intervals over a long recording window, brief bursts of rapid PV activity may be averaged out, thereby diluting transient acceleration and potentially obscuring clinically relevant PV firing. These limitations may provide an explanation for the discrepancies observed in the literature.^20,21^ The FARS_10_ measurement, excluding by definition continuously fragmented signal and being able to capture transient acceleration, overcomes the limitations of previous AF-CL approaches. This method demonstrates a high degree of reproducibility in different atrial locations and also a high degree of inter-observer reproducibility.^12^

The present study provides additional support for the method and expands the applicability of this approach to a mixed large cohort of AF patients with paroxysmal and persistent AF.

### Pulmonary vein isolation responders vs. pulmonary vein isolation non-responders

A large number of studies have assessed the role of clinical predictors in the effectiveness of AF ablation with variable outcomes.^4^ Few studies have assessed the relationship between AF-CL and ablation outcomes with conflicting results. Pascale *et al.*^20^ reported that the ratio of fastest PV and LAA-CL was a predictor of acute termination, the need for limited adjunctive substrate ablation, and good long-term outcome. Prabhu *et al.*^21^ reported that the rapidity of PV activity in absolute value or as ratio compared to LAA-CL was not predictive of multi-procedural ablation success. The methodology of AF-CL measurement over a longer window and including fragmented signals and double potentials, as well as the lack of standardized PVI-only ablation approach, may explain the discrepant findings. In the current and the previously reported studies from our group, we confirm that the utilization of the novel PV-FARS_10_ measurement method reliably distinguishes persistent AF patients in whom PVI alone was successful. Specifically, patients with fast PV-FARS_10_ experienced lower AF recurrences (PVI responders) as compared to patients with slow PV-FARS_10_ (PVI non-responders). The current study not only reinforces the same finding but also confirms the outcome with a longer follow-up period and larger study population. This better stratification of AF patients may result in the development of customized ablation strategies, which enables the use of additional ablations only in PVI non-responders. In the absence of tailored ablation strategies, adding empirical additional ablation, as performed in prior trials,^7^ may lead to new arrhythmias and may have a counterproductive effect. The present study has also confirmed the finding that PV-FARS_10_ is a strong predictor of AF recurrence in paroxysmal AF. Furthermore, for the first time, shortest PV-FARS_10_ has been shown to be a strong predictor of AF recurrence also in a mixed patient population, including individuals with both paroxysmal and persistent AF. Our findings support the hypothesis that transiently fastest regularized regions could act as triggers and/or drivers. In our cohort, the PV/LAA-FARS_10_ ratio showed a borderline association with AF recurrence, suggesting potential incremental prognostic value that warrants confirmation in larger, adequately powered populations. However, a causal relationship between fast PV-CL activity and AF mechanism is yet to be demonstrated. A healthier substrate could allow faster conduction also into the PVs, and patients with fast PV-CL may have a lower recurrence rate due to the healthier atrium. In contrast, an underlying structural atrial substrate, known to have a negative outcome of PVI-only approach, could be linked to slow PV-CL.^14^ Alternatively, repolarization heterogeneities with short PV and long surrounding antral refractory periods may also be the mechanism of arrhythmogenicity and AF initiation and maintenance in patients with fast PV-CL. However, irrespective of the mechanism, the consistent results from our studies suggest that PV-FARS_10_ is a powerful tool to classify patients according to PV responders or PVI non-responders, rather than relying on the current clinical AF pattern of paroxysmal and persistent AF. In support of this hypothesis, in the current study, we did not observe a significant difference in shortest PV-FARS_10_ between persistent and paroxysmal AF patients.

### Pulmonary vein activity in patients with low-voltage zones

The presence of spontaneous LVZ is thought to correlate with more extensive substrate and was associated with high AF recurrence following PVI.^22^ In our study, the presence of spontaneous LVZ was not associated with worse outcomes; however, there was a statistically significant lower rate of LVZ among patients with fast PV-FARS_10_.

## Limitations

FARS_10_ assessment requires manual calliper measurements and visual identification of repetitive similar morphology intervals and therefore may be operator dependent. Although reproducibility has been previously reported, inter-observer (and intra-observer) variability was not reassessed in the present cohort; thus, external validity across operators/centres warrants confirmation. In the future, automatic assessment of the FARS_10_ may overcome this limitation. By excluding complex electrograms, FARS_10_ may underestimate very rapid PV activity that rapidly becomes continuously fragmented and non-analysable, potentially biasing measurements towards longer values or preventing quantification in patients with more advanced atrial remodelling. The discontinuation of ADDs only on the day of PVI may significantly affect FARS_10_ measurements and the proposed cut-off value for distinguishing fast and slow PV-CL. Indeed, the cut-off value exhibits low specificity across all subpopulations. In spite of novel ablation technologies, PV reconnection occurs and may mask predictors of PVI-only approach. The absence of continuous rhythm monitoring for the majority of the patients during follow-up is another limitation of the study and may lead to the underestimation of AF recurrence. The small study population should also be acknowledged. Furthermore, because patients undergoing adjunctive substrate modification were excluded, the cohort may have been enriched for less advanced atrial remodelling, potentially affecting acute AF termination and long-term recurrence. Low-voltage zone assessment during AF was performed using a bipolar voltage cut-off of 0.24 mV, a threshold that remains debated in the literature.^23^ Finally, in patients undergoing Cryo, PV and LAA-CL measurements were obtained using the Achieve catheter; although only high-quality, stable recordings meeting predefined criteria were analysed, catheter-related differences in electrogram resolution and contact may have introduced residual variability in CL assessment.

## Conclusion

Our study confirms that in AF patients and in patients with persistent and paroxysmal AF, the shortest PV-FARS_10_ is independently associated with AF recurrence following PVI-only approach. The value of ≤155 ms shortest PV-FARS_10_ is capable of identifying PVI responder patients with favourable long-term success rate after PVI-only ablation approach in paroxysmal AF, in persistent AF, and in an overall AF population. Patients with fast PV-FARS_10_ (≤155 ms) exhibit a lower rate of AF recurrences compared to those with slow PV-FARS_10_ (>155 ms). Additionally, no significant difference was found in PV-FARS_10_ between persistent and paroxysmal AF patients, although paroxysmal AF patients more frequently have the shortest PV-FARS_10_ ≤ 155 ms.

## Supplementary Material

euag033_Supplementary_Data

## Data Availability

The data underlying this article cannot be shared publicly due to ethical and privacy restrictions. De-identified data may be shared on reasonable request to the corresponding author.
