# Supplementary material for: Shortest pulmonary vein atrial fibrillation cycle length identifies pulmonary vein isolation responders beyond clinical atrial fibrillation pattern: the FARS-AF II study
Source: Europace. 2026 Feb 23;28(2):euag033. doi: 10.1093/europace/euag033 (PMC12964357; doi:10.1093/europace/euag033)
Supplement: euag033_Supplementary_Data [file euag033_supplementary_data.zip › Supplementary_Data.docx]

**SUPPLEMENTARY DATA**

INCLUSION AND EXCLUSION CRITERIA

Paroxysmal AF patients

Inclusion criteria : 1) diagnosis of paroxysmal AF in patients undergoing first-AF ablation, 2) AF present at baseline or sustained AF inducible at the beginning of the procedure.

Exclusion criteria : 1) deployment of additional left atrial focal or linear ablations in addition to PVI; 2) induction of only organized arrhythmias such as atypical left atrial flutter or atrial tachycardia; 3) participation in a competing research study.

The need for adjunctive ablation beyond PVI was determined at the operator’s discretion based on intra-procedural findings (electroanatomical mapping, induction of organized atrial arrhythmias). Therefore, eligibility for inclusion was finalized intra-procedurally.

Successful induction of sustained AF was defined as AF persisting longer than 5 minutes after the induction.

Persistent AF patients

Inclusion criteria: 1) diagnosis of persistent AF in patients undergoing first-AF ablation, 2) AF present at baseline or sustained AF inducible at the beginning of the procedure.

Exclusion criteria : 1) deployment of additional left atrial focal or linear ablations in addition to PVI; 2) induction of only organized arrhythmias such as atypical left atrial flutter or atrial tachycardia; 3) participation in a competing research study.

The need for adjunctive ablation beyond PVI was determined at the operator’s discretion based on intra-procedural findings (electroanatomical mapping, induction of organized atrial arrhythmias). Therefore, eligibility for inclusion was finalized intra-procedurally.

Successful induction of sustained AF was defined as AF persisting longer than 5 minutes after the induction.

Selection considerations

Because sustained AF induction and PV-FARS_10_ acquisition were not feasible in all otherwise eligible cases due to workflow constraints, participation in concomitant studies, and potential operator-related factors, selection bias cannot be excluded.

FARS_10_ MEASUREMENTS

All cycle lengths measurements were obtained manually using electronic calipers. The average of 10 consecutive Fastest Atrial Repetitive Similar morphology signal cycle lengths (FARS_10_) in a 1-minute observational window was measured. FARS signals were defined as having all of the following characteristics: (I) discrete signal with a duration less <50% of CL; (II) repetitive similar morphological characteristics; (III) during 1-minute observation the duration of the fastest consecutive 10 intervals is measured and divided by 10 to calculate the mean FARS_10_-CL in msec.

Continuously fragmented or changing morphology complex signals were excluded from FARS_10_ measurements. Double potentials with different signal morphology of the two closely coupled components were also excluded. In the PVs, if signals show continuous fragmentation or closely coupled double potentials with different signal morphology, the observation window was prolonged and/or a more distal or proximal PV recording site with regularized signals was searched.

ABLATION PROCEDURE

All procedures were performed under general anesthesia or conscious sedation. Vitamin K antagonists were maintained at a target INR between 2 and 3, whereas direct oral anticoagulants were discontinued on the day of the procedure and resumed on the same day. After groin puncture, intravenous heparin was administered to maintain an activated clotting time between 300 and 350 seconds. The discontinuation of anti-arrhythmic drugs prior to the procedure was left to the operators’ discretion.

The catheter ablation procedure employed radiofrequency energy for patients with persistent AF, while both radiofrequency energy and cryoballoon were utilized for patients with paroxysmal AF.

Radiofrequency Ablation

LA geometry (FAM) was constructed with a 20-pole Lasso catheter with 1 mm electrode size and 2 mm interelectrode spacing (Biosense Webster) using the CARTO3 electroanatomical mapping system (Biosense Webster). Automatic mapping algorithm (Confidence module, Biosense Webster) was used with the following predefined settings: in SR atrial CL ± 10% ms, mapping catheter position stability 1 or 2mm, LAT stability in sinus rhythm 3ms, density maximum or 1mm. In AF, 400ms window of interest was used excluding the QRS complex and LAT stability was turned off. Irrigated tip ablation catheter with contact force-sensing technology (Thermocool SmartTouch catheter, Biosense Webster, Diamond Bar, California) was used for ablation. RF lesions were placed in temperature-limited power control mode. The ablation was guided by automatic ablation annotation and minimum force-time integral and, later, ablation index values (VisiTag®, Biosense Webster, Diamond Bar, CA, USA), together with local electrogram attenuation and impedance changes. RF applications were performed with a target numeric contact force value of > 10g and ≤ 5 mm inter-lesion distance. Wide area bilateral circumferential PV isolation (PVI) was performed in the antrum of ipsilateral PVs with > 1cm distance from the PV ostium except for the left superior PV and the ridge of the left atrial appendage. Entry and exit block of the PVs were assessed with and without intravenous adenosine and if needed touch up applications were applied at the gap sites to achieve block.

Cryoballoon ablation

Cryoballoon ablation was performed with a second-generation Cryoballoon ablation. Following single transseptal puncture a steerable sheath (Flex-Cath®, Medtronic Inc) was advanced into the left atrium. A multipolar circular mapping catheter (Achieve, Medtronic Inc.) was placed in the pulmonary veins for monitoring PV potentials before, during and following cryoablation. The 28 mm (Arctic Front Advanced, Medtronic Inc.) cryoballoon catheter was used for cryoablation. PV occlusion was verified with contrast injection and freezing time was chosen between 180 and 240s with monitoring time to PV isolation and nadir temperatures. A bonus of 180s was administered if time to isolation was > 60s or minimal balloon temperature was above – 40°C. Ablation of the right PVs were performed under continuous phrenic nerve pacing.

ANTI-ARRHYTHMIC MEDICATIONS

Discontinuation of anti-arrhythmic drugs (AADs) before the procedure was left to the operators’ discretion; however, all patients stopped AAD therapy on the day of the procedure. At baseline, 51 patients (23.3%) were receiving flecainide, 54 (24.7%) sotalol, and 42 (19.2%) amiodarone. Post-ablation AADs therapy during the 3-month blanking period was prescribed at the treating physician’s discretion; thereafter, AADs were routinely discontinued.

INDUCTION PROTOCOL

After CS catheter placement into the coronary sinus, induction was attempted with atrial burst pacing (20 beats), starting from 300 ms and decreasing cycle length (10 ms) until atrial refractory period. Once atrial refractoriness has been assessed, up to five burst were delivered 20 ms slower than atrial refractoriness. If AF was not induced with CS pacing, the same induction protocol was be performed from the circular mapping catheter inside the pulmonary veins. Sustained AF was defined as AF lasting for at least 5 minutes after induction.

| Table S1: Baseline characteristics of the study population, based on shortest PV-FARS_10_ | | | | |
| --- | --- | --- | --- | --- |
| Characteristic | **Total**  N = 219 | **Fast PV**  **(≤ 155ms)**  N = 106 | **Slow PV**  **(> 155ms)**  N = 113 | p value |
| Age, years | **61.8 ± 11.2** | **59.5 ± 11.4** | **64.0 ± 10.6** | **0.003** |
| Female Sex | **55 (25.1%)** | **19 (17.9%)** | **36 (31.9%)** | **0.02** |
| Hypertension | **111 (50.7%)** | **46 (43.4%)** | **65 (57.5%)** | **0.04** |
| Diabetes | 28 (12.8%) | 13 (12.3%) | 15 (13.3%) | 0.84 |
| BMI, kg/m^2^ | **28.5 ± 4.93** | **27.8 ± 4.4** | **29.1 ± 5.3** | **0.05** |
| Coronary Artery Disease | 34 (15.5%) | 13 (12.3%) | 21 (18.6%) | 0.26 |
| CHA_2_DS_2_-VA score | **2 (0 – 3)** | **1 (0 – 2)** | **2 (1 – 3)** | **< 0.001** |
| Obstructive sleep apnea | 33 (15.1%) | 14 (13.2%) | 19 (16.8%) | 0.57 |
| Ejection Fraction, % | 56.9 ± 9.3 | 57.9 ± 8.4 | 56.0 ± 10.0 | 0.15 |
| LAVI, mL/m^2^ | 39.5 ± 12.4 | 38.0 ± 12.9 | 40.8 ± 11.8 | 0.09 |
| Years since 1^st^ AF episode | **2.1 (0.8 – 5.3)** | **1.7 (0.6 – 4.5)** | **2.9 (1.1 – 5.7)** | **0.03** |
| Patients on Flecainide | 51 (23.3%) | 19 (17.9%) | 32 (28.3%) | 0.08 |
| Patients on Sotalol | **54 (24.7%)** | **34 (32.1%)** | **20 (17.7%)** | **0.02** |
| Patients on Amiodarone | **42 (19.2%)** | **9 (8.5%)** | **33 (29.2%)** | **< 0.001** |
| Paroxysmal AF | **70 (32%)** | **43 (40.6%)** | **27 (23.9%)** | **0.009** |
| Persistent AF | **149 (68%)** | **63 (59.4%)** | **86 (76.1%)** | **0.009** |
| *Procedure-related information* | | | | |
| AF termination during procedure | **71 (32.6%)** | **42 (40%)** | **29 (25.7%)** | **0.03** |
| Continuous variables are shown as Mean ± Standard Deviation (SD) or Median and (Inter Quartile Range) (IQR). Discrete variables are presented as numbers and percentages (%).  Abbreviation List: BMI (Body Mass Index), LAVI (Left Atrial Volume Index), PV (Pulmonary Vein), FARS_10_ (10 consecutive Fastest Atrial Repetitive Similar morphology signal), CL (Cycle Length). | | | | |

| Table S2: Baseline characteristics of the study population stratified by AF/AFL/AT recurrence during follow-up | | | | |
| --- | --- | --- | --- | --- |
| Characteristic | **Total**  N = 219 | **AF/AFL/AT recurrence** N = 72 | **AF/AFL/AT no recurrence** N = 147 | p value |
| Age, years | 61.8 ± 11.2 | 63.6 ± 10.3 | 60.9 ± 11.5 | 0.10 |
| Female Sex | 55 (25.1%) | 22 (30.6%) | 33 (22.4%) | 0.24 |
| Hypertension | 111 (50.7%) | 40 (55.6%) | 71 (48.3%) | 0.32 |
| Diabetes | 28 (12.8%) | 9 (12.5%) | 19 (12.9%) | 1 |
| BMI, kg/m^2^ | 28.5 ± 4.93 | 28.6 ± 4.5 | 28.4 ± 5.1 | 0.80 |
| Coronary Artery Disease | 34 (15.5%) | 12 (16.7%) | 22 (15%) | 0.84 |
| CHA_2_DS_2_-VA score | 2 (0 – 3) | 2 (1 – 3) | 1 (0 – 3) | 0.19 |
| Obstructive sleep apnea | 33 (15.1%) | 13 (18.1%) | 20 (13.6%) | 0.42 |
| Ejection Fraction | 56.9 ± 9.3 | 55.8 ± 9.1 | 57.4 ± 9.3 | 0.24 |
| LAVI, mL/m^2^ | **39.5 ± 12.4** | **42.8 ± 12.7** | **37.9 ± 11.9** | **0.007** |
| Years since 1^st^ AF episode | 2.1 (0.8 – 5.3) | 2.2 (0.8 – 5.8) | 2.1 (0.8 – 5.2) | 0.94 |
| Patients on Flecainide | 51 (23.3%) | 18 (25%) | 33 (22.4%) | 0.73 |
| Patients on Sotalol | 54 (24.7%) | 16 (22.2%) | 38 (25.9%) | 0.62 |
| Patients on Amiodarone | 42 (19.2%) | 19 (26.4%) | 23 (15.6%) | 0.07 |
| Paroxysmal AF | **70 (32%)** | **12 (16.7%)** | **58 (39.5%)** | **< 0.001** |
| Persistent AF | **149 (68%)** | **60 (83.3%)** | **89 (60.5%)** | **< 0.001** |
| *Procedure-related information* | | | | |
| AF termination during procedure | **71 (32.6%)** | **14 (19.7%)** | **57 (38.8%)** | **0.005** |
| Fast PV (≤ 155ms) | **106 (48.4%)** | **20 (27.8%)** | **86 (58.5%)** | **< 0.001** |
| Continuous variables are shown as Mean ± Standard Deviation (SD) or Median and (Inter Quartile Range) (IQR). Discrete variables are presented as numbers and percentages (%).  Abbreviation List: BMI (Body Mass Index), LAVI (Left Atrial Volume Index), FARS_10_ (10 consecutive Fastest Atrial Repetitive Similar morphology signal), PV (Pulmonary Vein). | | | | |

| Table S3: Comparison of FARS10 measurements in left and right atrial structures between patients with paroxysmal and persistent AF | | | | |
| --- | --- | --- | --- | --- |
| Characteristic | **Total**  N = 219 | **Persistent AF**  N = 149 | **Paroxysmal AF**  N = 70 | p value |
| TOTAL n = 219 | | | | |
| LSPV FARS_10_, ms | 170.8 ± 40.6 | 170.5 ± 37.2 | 171.4 ± 47.1 | 0.89 |
| LIPV FARS_10_, ms | 173.2 ± 40.4 | 173.3 ± 37.9 | 173.0 ± 45.6 | 0.96 |
| RIPV FARS_10_, ms | 180.6 ± 42.3 | 180.7 ± 37.6 | 180.4 ± 51.5 | 0.95 |
| RSPV FARS_10_, ms | 179.3 ± 41.2 | 179.3 ± 39.7 | 178.6 ± 44.6 | 0.85 |
| Shortest PV FARS_10_, ms | 162.1 ± 41.0 | 164.2 ± 38.7 | 157.5 ± 45.4 | 0.25 |
| CS FARS_10_, ms | 191.1 ± 36.9  (180/219) | 187.9 ± 34.6 (114/149) | 196.7 ± 40.3 (66/70) | 0.12 |
| LAA FARS_10_, ms | 183.8 ± 36.0  (185/219) | 182.2 ± 34.5 (139/149) | 188.6 ± 40.0 (46/70) | 0.29 |
| RAA FARS_10_, ms | 181.4 ± 33.2 (134/219) | 180.3 ± 32.3 (101/149) | 184.8 ± 35.9 (33/70) | 0.50 |
| SVC FARS_10_, ms | 214.7 ± 47.2 (58/219) | 214.3 ± 50.1 (43/149) | 216.0 ± 39.7 (15/70) | 0.90 |
| FAST PV ≤ 155 n = 106 | | | | |
| LSPV FARS_10_, ms | 145.6 ± 27.6 | 143.7 ± 25.0 | 148.3 ± 31.1 | 0.39 |
| LIPV FARS_10_, ms | 147.2 ± 23.6 | 144.4 ± 21.9 | 151.5 ± 25.6 | 0.14 |
| RIPV FARS_10_, ms | 155.6 ± 26.3 | 155.4 ± 27.1 | 155.9 ± 25.1 | 0.93 |
| RSPV FARS_10_, ms | 153.6 ± 25.7 | 153.4 ± 27.3 | 153.9 ± 23.4 | 0.92 |
| Shortest PV FARS_10_, ms | 131.6 ± 19.0 | 131.3 ± 19.2 | 131.8 ± 19.0 | 0.90 |
| CS FARS_10_, ms | 177.7 ± 28.3  (90/106) | 173.8 ± 29.4  (48/63) | 182.2 ± 26.8  (42/43) | 0.16 |
| LAA FARS_10_, ms | 171.2 ± 28.3  (88/106) | 168.7 ± 28.6  (60/63) | 176.5 ± 27.3  (28/43) | 0.23 |
| RAA FARS_10_, ms | 166.1 ± 23.8  (63/106) | 163.3 ± 24.3  (44/63) | 172.4 ± 22.0  (19/43) | 0.17 |
| SVC FARS_10_, ms | **199.9 ± 40.1**  **(31/106)** | **190.0 ± 32.9**  **(21/63)** | **220.7 ± 47.4**  **(10/43)** | **0.04** |
| SLOW PV > 155 n = 113 | | | | |
| LSPV FARS_10_, ms | **195.6 ± 35.7** | **191.1 ± 31.5** | **209.4 ± 44.5** | **0.02** |
| LIPV FARS_10_, ms | 198.3 ± 37.2 | 195.3 ± 32.4 | 207.9 ± 49.5 | 0.14 |
| RIPV FARS_10_, ms | 203.6 ± 41.3 | 199.3 ± 33.2 | 217.1 ± 59.1 | 0.06 |
| RSPV FARS_10_, ms | **203.7 ± 38.3** | **199.5 ± 36.0** | **216.5 ± 42.9** | **0.04** |
| Shortest PV FARS_10_, ms | 190.7 ± 35.0 | 188.3 ± 30.9 | 198.3 ± 45.6 | 0.19 |
| CS FARS_10_, ms | **204.5 ± 39.6 (90/113)** | **198.1 ± 34.6**  **(66/86)** | **222.2 ± 47.4**  **(24/27)** | **0.01** |
| LAA FARS_10_, ms | 195.2 ± 38.5 (97/113) | 192.4 ± 35.3 (79/86) | 207.5 ± 49.4 (18/27) | 0.13 |
| RAA FARS_10_, ms | 195.1 ± 34.4 (71/113) | 193.4 ± 31.8 (57/86) | 201.6 ± 44.4 (14/27) | 0.43 |
| SVC FARS_10_, ms | 231.8 ± 49.7 (27/113) | 237.5 ± 53.1 (22/86) | 206.6 ± 17.2 (5/27) | 0.21 |
| Continuous variables are shown as Mean ± Standard Deviation (SD) or Median and (Inter Quartile Range) (IQR). Discrete variables are presented as numbers and percentages (%).  Abbreviation List: LSPV (Left Superior Pulmonary Vein), LIPV ( Left Inferior Pulmonary Vein), RIPV (Right Inferior Pulmonary Vein), RSPV (Right Superior Pulmonary Vein), CS (Coronary Sinus), LAA (Left Atrial Appendage), RAA (Right Atrial Appendage), SVC (Superior Vena Cava). | | | | |

| Table S4: FARS_10_ measurements in the PVs and different atrial structures in patients with fast vs slow PVs | | | | |
| --- | --- | --- | --- | --- |
| Characteristic | **Total**  N = 219 | **Fast PV**  **(**≤ **155 ms)**  N = 106 | **Slow PV**  **(> 155ms)**  N = 113 | p value |
| LSPV FARS_10_, ms | **170.8 ± 40.6** | **145.6 ± 27.6** | **195.6 ± 35.7** | **-** |
| LIPV FARS_10_, ms | **173.2 ± 40.4** | **147.2 ± 23.6** | **198.3 ± 37.2** | **-** |
| RIPV FARS_10_, ms | **180.6 ± 42.3** | **155.6 ± 26.3** | **203.6 ± 41.3** | **-** |
| RSPV FARS_10_, ms | **179.3 ± 41.2** | **153.6 ± 25.7** | **203.7 ± 38.3** | **-** |
| Shortest PV FARS_10_, ms | **162.1 ± 41.0** | **131.6 ± 19.0** | **190.7 ± 35.0** | **-** |
| CS FARS_10_, ms | **191.1 ± 36.9**  **(180/219)** | **177.7 ± 28.3**  **(90/106)** | **204.5 ± 39.6 (90/113)** | **-** |
| LAA FARS_10_, ms | **183.8 ± 36.0 (185/219)** | **171.2 ± 28.3 (88/106)** | **195.2 ± 38.5 (97/113)** | **-** |
| RAA FARS_10_, ms | **181.4 ± 33.2 (134/219)** | **166.1 ± 23.8 (63/106)** | **195.1 ± 34.4 (71/113)** | **-** |
| SVC FARS_10_, ms | **214.7 ± 47.2 (58/219)** | **199.9 ± 40.1 (31/106)** | **231.8 ± 49.7 (27/113)** | **-** |
| Continuous variables are shown as Mean ± Standard Deviation (SD) or Median and (Inter Quartile Range) (IQR). Discrete variables are presented as numbers and percentages (%).  Abbreviation List: LSPV (Left Superior Pulmonary Vein), LIPV ( Left Inferior Pulmonary Vein), RIPV (Right Inferior Pulmonary Vein), RSPV (Right Superior Pulmonary Vein), CS (Coronary Sinus), LAA (Left Atrial Appendage), RAA (Right Atrial Appendage), SVC (Superior Vena Cava). | | | | |

| Table S5: Comparison of right and left atrial FARS_10_ between patients with and without AF/AFL/AT recurrence | | | | |
| --- | --- | --- | --- | --- |
| Characteristic | **Total**  N = 219 | **AF/AFL/AT recurrence** N = 72 | **AF/AFL/AT no recurrence**  N = 147 | p value |
| TOTAL n = 219 | | | | |
| LSPV FARS_10_, ms | **170.8 ± 40.6** | **184.6 ± 47.8** | **164.1 ± 34.8** | **< 0.001** |
| LIPV FARS_10_, ms | **173.2 ± 40.4** | **191.3 ± 47.1** | **164.6 ± 33.7** | **< 0.001** |
| RIPV FARS_10_, ms | **180.6 ± 42.3** | **196.4 ± 54.2** | **172.9 ± 32.5** | **< 0.001** |
| RSPV FARS_10_, ms | **179.3 ± 41.2** | **196.1 ± 50.3** | **170.9 ± 32.8** | **< 0.001** |
| Shortest PV FARS_10_, ms | **162.1 ± 41.0** | **178.9 ± 47.3** | **153.8 ± 34.8** | **< 0.001** |
| CS FARS_10_, ms | **191.1 ± 36.9**  **(180/219)** | **202.3 ± 42.4 (54/72)** | **186.3 ± 33.3 (126/147)** | **0.007** |
| LAA FARS_10_, ms | **183.8 ± 36.0**  **(185/219)** | **192.1 ± 41.9 (63/72)** | **179.5 ± 31.8 (122/147)** | **0.02** |
| Shortest PV FARS_10_/LAA-FARS_10_ (%) | 89.3 ± 19.1  (185/219) | 92.7 ± 17.0  (63/72) | 87.5 ± 19.9  (122/147) | 0.08 |
| RAA FARS_10_, ms | **181.4 ± 33.2 (134/219)** | **191.9 ± 38.7 (47/72)** | **175.7 ± 28.3 (87/147)** | **0.006** |
| LAA/RAA FARS_10_ ratio (%) | 90.9 ± 27.8  (134/219) | 91.3 ± 25.4  (47/72) | 90.7 ± 29.1  (87/147) | 0.91 |
| SVC FARS_10_, ms | 214.7 ± 47.2 (58/219) | 223.4 ± 48.5 (13/72) | 212.3 ± 47.2 (45/147) | 0.46 |
| PERSISTENT AF n = 149 | | | | |
| LSPV FARS_10_, ms | **170.5 ± 37.2** | **179.5 ± 42.5** | **164.4 ± 31.9** | **0.02** |
| LIPV FARS_10_, ms | **173.3 ± 37.9** | **185.2 ± 39.9** | **165.1 ± 34.4** | **0.002** |
| RIPV FARS_10_, ms | 180.7 ± 37.6 | 187.8 ± 42.8 | 175.8 ± 32.9 | 0.06 |
| RSPV FARS_10_, ms | **179.3 ± 39.7** | **189.5 ± 46.9** | **172.5 ± 31.9** | **0.01** |
| Shortest PV FARS_10_, ms | **164.2 ± 38.7** | **173.6 ± 42.3** | **157.9 ± 34.9** | **0.01** |
| CS FARS_10_, ms | 187.9 ± 34.6 (114/149) | 194.3 ± 34.9 (43/60) | 184.0 ± 34.0 (71/89) | 0.12 |
| LAA FARS_10_, ms | 182.2 ± 34.5 (139/149) | 186.9 ± 36.6 (57/60) | 178.9 ± 32.8 (82/89) | 0.18 |
| Shortest PV FARS_10_/LAA-FARS_10_ (%) | 91.1 ± 18.6  (139/149) | 93.0 ± 17.2  (57/60) | 89.7 ± 19.4  (82/89) | 0.31 |
| RAA FARS_10_, ms | **180.3 ± 32.3 (101/149)** | **188.6 ± 36.4 (42/60)** | **174.5 ± 27.9 (59/89)** | **0.03** |
| LAA/RAA FARS_10_ ratio (%) | 93.3 ± 23.2  (101/149) | 92.7 ± 22.5  (42/60) | 93.7 ± 23.9  (59/60) | 0.82 |
| SVC FARS_10_, ms | 214.3 ± 50.1 (43/149) | 224.8 ± 52.9 (11/60) | 210.7 ± 49.4 (32/89) | 0.42 |
| PAROXYSMAL AF n = 70 | | | | |
| LSPV FARS_10_, ms | **171.4 ± 47.1** | **211.3 ± 65.9** | **163.8 ± 38.9** | **0.002** |
| LIPV FARS_10_, ms | **173.0 ± 45.6** | **235.4 ± 70.7** | **163.9 ± 32.8** | **< 0.001** |
| RIPV FARS_10_, ms | **180.4 ± 51.5** | **246.6 ± 84.0** | **168.3 ± 31.6** | **< 0.001** |
| RSPV FARS_10_, ms | **178.6 ± 44.6** | **235.6 ± 54.7** | **168.4 ± 34.1** | **< 0.001** |
| Shortest PV FARS_10_, ms | **157.5 ± 45.4** | **205.3 ± 62.8** | **147.6 ± 34.0** | **< 0.001** |
| CS FARS_10_, ms | **196.7 ± 40.3 (66/70)** | **233.4 ± 55.3 (11/12)** | **189.4 ± 32.5 (55/58)** | **< 0.001** |
| LAA FARS_10_, ms | **188.6 ± 40.0 (46/70)** | **241.3 ± 59.7 (6/12)** | **180.7 ± 30.0 (40/58)** | **< 0.001** |
| Shortest PV FARS_10_/LAA-FARS_10_ (%) | 83.9 ± 19.7  (46/70) | 90.3 ± 15.0  (6/12) | 83.0 ± 20.3  (40/58) | 0.40 |
| RAA FARS_10_, ms | **184.8 ± 35.9 (33/70)** | **220.4 ± 50.2 (5/12)** | **178.4 ± 29.6 (28/58)** | **0.01** |
| LAA/RAA FARS_10_ ratio (%) | 83.7 ± 38.1 (33/70) | 79.6 ± 45.1  (5/12) | 84.5 ± 37.6  (25/58) | 0.79 |
| SVC FARS_10_, ms | 216.0 ± 39.7 (15/70) | 215.5 ± 6.3 (2/12) | 216.1 ± 42.8 (13/58) | 0.98 |
| Continuous variables are shown as Mean ± Standard Deviation (SD) or Median and (Inter Quartile Range) (IQR). Discrete variables are presented as numbers and percentages (%).  Abbreviation List: : LSPV (Left Superior Pulmonary Vein), LIPV ( Left Inferior Pulmonary Vein), RIPV (Right Inferior Pulmonary Vein), RSPV (Right Superior Pulmonary Vein), CS (Coronary Sinus), LAA (Left Atrial Appendage), RAA (Right Atrial Appendage), SVC (Superior Vena Cava). | | | | |

| Table S6: Comparison of clinical outcomes during follow-up between paroxysmal and persistent AF | | | | | |
| --- | --- | --- | --- | --- | --- |
| Characteristic | **Total**  N = 219 | **Persistent AF**  N = 149 | | **Paroxysmal AF**  N = 70 | p value |
| AF/AFL/AT Recurrence | **72 (32.9%)** | **60 (40.3%)** | | **12 (17.1%)** | **< 0.001** |
| Redo | **27 (12.3%)** | **24 (16.1%)** | | **3 (4.3%)** | **0.01** |
| PV reconnection at Redo | 9 (33.3%) | 8 (33.3%) | | 1 (33.3%) | 1 |
| AF/AFL/AT Recurrence after Redo | 7/18 (38.9%) | 7/18 (38.9%) | | / | - |
| Continuous variables are shown as Mean ± Standard Deviation (SD) or Median and (Inter Quartile Range) (IQR). Discrete variables are presented as numbers and percentages (%).  Abbreviation List: AF (Atrial Fibrillation), AFL (Atrial Flutter), AT (Atrial Tachycardia). | | |  | | |

| Table S7: Comparison of FARS_10_ measurements depending on anti-arrhythmic drug therapy | | | |
| --- | --- | --- | --- |
| Characteristic | **AADs**  **(n = 147)** | **No AADs**  **(n = 75)** | p value |
| AF termination – no.(%) | 48 (32.6%) | 23 (30.7%) | 0.76 |
| LSPV FARS_10_, ms (mean ± SD) | **176.4 ± 44.8** | **160.5 ± 28.8** | **0.006** |
| LIPV FARS_10_, ms | **180.4 ± 44.1** | **159.7 ± 28.1** | **< 0.001** |
| RIPV FARS_10_, ms | **187.3 ± 43.2** | **167.9 ± 37.9** | **0.002** |
| RSPV FARS_10_, ms | **186.1 ± 45.3** | **166.7 ± 28.5** | **0.001** |
| Shortest PV FARS_10_, ms | **168.4 ± 45.9** | **149.9 ± 25.6** | **0.001** |
| CS FARS_10_, ms | **198.1 ± 39.1** | **178.4 ± 28.8** | **< 0.001** |
| LAA FARS_10_, ms | **191.2 ± 37.6** | **169.5 ± 27.8** | **< 0.001** |
| RAA FARS_10_, ms | **188.9 ± 36.3** | **168.4 ± 21.6** | **< 0.001** |
| SVC FARS_10_, ms | 218.2 ± 50.7 | 208.7 ± 40.8 | 0.46 |
| Continuous variables are shown as Mean ± Standard Deviation (SD). Discrete variables are presented as numbers and percentages (%).  Among patients in the “AADs” 51 (23.3%) patients were under Flecainide, 54 (24.7%) under Sotalol and 42 (19.2%) under Amiodarone.    Abbreviation List: LSPV (Left Superior Pulmonary Vein), LIPV ( Left Inferior Pulmonary Vein), RIPV (Right Inferior Pulmonary Vein), RSPV (Right Superior Pulmonary Vein), CS (Coronary Sinus), LAA (Left Atrial Appendage), RAA (Right Atrial Appendage), SVC (Superior Vena Cava), AAD (Anti-arrhythmic Drug) | | | |

| Table S8: Comparison of right and left atrial FARS_10_ between patients with spontaneous and induced AF | | | | |
| --- | --- | --- | --- | --- |
| Characteristic | **Total**  N = 219 | **Spontaneous AF**  N = 134 | **Induced AF**  N = 85 | p value |
| TOTAL n = 219 | | | | |
| LSPV FARS_10_, ms | 170.8 ± 40.6 | 171.9 ± 40.3 | 169.1 ± 41.1 | 0.62 |
| LIPV FARS_10_, ms | 173.2 ± 40.4 | 175.7 ± 39.6 | 168.9 ± 41.6 | 0.25 |
| RIPV FARS_10_, ms | 180.6 ± 42.3 | 181.4 ± 40.6 | 179.4 ± 45.2 | 0.74 |
| RSPV FARS_10_, ms | 179.3 ± 41.2 | 182.0 ± 42.5 | 175.0 ± 38.9 | 0.23 |
| Shortest PV FARS_10_, ms | **162.1 ± 41.0** | **166.5 ± 41.6** | **155.0 ± 39.1** | **0.04** |
| CS FARS_10_, ms | 191.1 ± 36.9  (180/219) | 187.9 ± 37.1  (109/134) | 196.0 ± 36.3  (71/85) | 0.15 |
| LAA FARS_10_, ms | 183.8 ± 36.0  (185/219) | 182.4 ± 36.4  (128/134) | 186.9 ± 35.2  (57/85) | 0.42 |
| RAA FARS_10_, ms | 181.4 ± 33.2 (134/219) | 180.6 ± 31.8  (99/134) | 183.9 ± 36.9  (35/85) | 0.61 |
| SVC FARS_10_, ms | 214.7 ± 47.2 (58/219) | 212.9 ± 49.9  (44/134) | 220.6 ± 38.6  (14/85) | 0.59 |
| AF termination – no.(%) | **71 (32.6%)** | **28 (20.9%)** | **43 (51.2%)** | **< 0.001** |
| Fast PV (≤ 155ms) | **106 (48.4%)** | **54 (40.3%)** | **52 (61.2%)** | **0.003** |
| Patients on Flecainide | 51 (23.3%) | 31 (23.1%) | 20 (23.5%) | 1.00 |
| Patients on Sotalol | 54 (24.7%) | 31 (23.1%) | 23 (27.1%) | 0.52 |
| Patients on Amiodarone | 42 (19.2%) | 31 (23.1%) | 11 (12.9%) | 0.08 |
|  |  |  |  |  |
| PERSISTENT AF | | | | |
|  | **Total**  N = 149 | **Spontaneous AF**  N = 125 | **Induced AF**  N = 24 |  |
| LSPV FARS_10_, ms | 170.5 ± 37.2 | 173.1 ± 36.8 | 157.0 ± 37.0 | 0.06 |
| LIPV FARS_10_, ms | **173.3 ± 37.9** | **176.6 ± 37.2** | **152.8 ± 37.1** | **0.01** |
| RIPV FARS_10_, ms | 180.7 ± 37.6 | 182.1 ± 38.9 | 173.0 ± 29.0 | 0.31 |
| RSPV FARS_10_, ms | **179.3 ± 39.7** | **182.6 ± 40.8** | **164.0 ± 29.2** | **0.04** |
| Shortest PV FARS_10_, ms | **164.2 ± 38.7** | **168.1 ± 38.6** | **144.2 ± 33.4** | **0.005** |
| CS FARS_10_, ms | 187.9 ± 34.6 (114/149) | 186.9 ± 35.6  (101/125) | 195.3 ± 25.0  (13/24) | 0.41 |
| LAA FARS_10_, ms | 182.2 ± 34.5 (139/149) | 180.3 ± 34.2  (121/125) | 195.1 ± 34.7  (18/24) | 0.09 |
| RAA FARS_10_, ms | 180.3 ± 32.3 (101/149) | 180.8 ± 32.1  (95/125) | 172.8 ± 37.4  (6/24) | 0.55 |
| SVC FARS_10_, ms | 214.3 ± 50.1 (43/149) | 214.2 ± 50.6  (42/125) | 220.0  (1/24) | - |
| AF termination – no.(%) | **37 (25%)** | **23 (18.4%)** | **14 (60.9%)** | **< 0.001** |
| Fast PV (≤ 155ms) | **63 (42.3%)** | **47 (37.6%)** | **16 (66.7%)** | **0.01** |
| Patients on Flecainide | 31 (20.8%) | 28(22.4%) | 3 (12.5%) | 0.41 |
| Patients on Sotalol | 38 (25.5%) | 30 (24.0%) | 8 (33.3%) | 0.44 |
| Patients on Amiodarone | 36 (24.2%) | 30 (24.0%) | 6 (25.0%) | 1.00 |
|  |  |  |  |  |
| PAROXYSMAL AF | | | | |
|  | **Total**  N = 70 | **Spontaneous AF**  N = 9 | **Induced AF**  N = 61 |  |
| LSPV FARS_10_, ms | 171.4 ± 47.1 | 155.9 ± 74.6 | 173.7 ± 41.9 | 0.28 |
| LIPV FARS_10_, ms | 173.0 ± 45.6 | 162.8 ± 68.7 | 174.5 ± 41.9 | 0.50 |
| RIPV FARS_10_, ms | 180.4 ± 51.5 | 170.5 ± 64.6 | 181.7 ± 49.9 | 0.56 |
| RSPV FARS_10_, ms | 178.6 ± 44.6 | 174.2 ± 63.5 | 179.2 ± 41.5 | 0.75 |
| Shortest PV FARS_10_, ms | 157.5 ± 45.4 | 145.3 ± 72.2 | 159.3 ± 40.6 | 0.39 |
| CS FARS_10_, ms | 196.7 ± 40.3 (66/70) | 200.7 ± 53.8  (8/9) | 196.1 ± 38.6  (58/61) | 0.76 |
| LAA FARS_10_, ms | **188.6 ± 40.0 (46/70)** | **218.8 ± 54.1**  **(7/9)** | **183.2 ± 35.2**  **(39/61)** | **0.03** |
| RAA FARS_10_, ms | 184.8 ± 35.9 (33/70) | 174.5 ± 26.6  (4/9) | 186.2 ± 37.1  (29/61) | 0.54 |
| SVC FARS_10_, ms | 216.0 ± 39.7 (15/70) | 185.5 ± 21.9  (2/9) | 220.7 ± 40.2  (13/61) | 0.26 |
| AF termination – no.(%) | 34 (48.6%) | 5 (55.6%) | 29 (47.5%) | 0.73 |
| Fast PV (≤ 155ms) | 43 (61.4%) | 7 (77.8%) | 36 (59.0%) | 0.46 |
| Patients on Flecainide | 20 (28.6%) | 3 (33.3%) | 17 (27.9%) | 0.71 |
| Patients on Sotalol | 16 (22.9%) | 1 (11.1%) | 15 (24.6%) | 0.67 |
| Patients on Amiodarone | 6 (8.6%) | 1 (11.1%) | 5 (8.2%) | 0.57 |
| Continuous variables are shown as Mean ± Standard Deviation (SD) or Median and (Inter Quartile Range) (IQR). Discrete variables are presented as numbers and percentages (%).  Abbreviation List: : AF (Atrial Fibrillation), LSPV (Left Superior Pulmonary Vein), LIPV ( Left Inferior Pulmonary Vein), RIPV (Right Inferior Pulmonary Vein), RSPV (Right Superior Pulmonary Vein), CS (Coronary Sinus), LAA (Left Atrial Appendage), RAA (Right Atrial Appendage), SVC (Superior Vena Cava). | | | | |

| Table S9: AF Termination during ablation and PV-FARS_10_ | | | | |
| --- | --- | --- | --- | --- |
| Characteristic | **Total**  N = 219 | **Persistent AF**  N = 149 | **Paroxysmal AF**  N = 70 | p value |
| AF termination during procedure | **71 (32.6%)** | **37 (25%)** | **34 (48.6%)** | **< 0.001** |
| AF termination during ablation at the fastest PV | **46/71 (64.8%)** | **18/37 (48.6%)** | **28/34 (82.4%)** | **0.006** |
| AF termination during ablation at the fastest PV (≤ 155ms) | **25/46 (54.3%)** | **6/18 (33.3%)** | **19/28 (67.9%)** | **0.03** |
| Continuous variables are shown as Mean ± Standard Deviation (SD) or Median and (Inter Quartile Range) (IQR). Discrete variables are presented as numbers and percentages (%).  Abbreviation List: AF (Atrial Fibrillation), FARS_10_ (10 consecutive Fastest Atrial Repetitive Similar morphology signal), PV (Pulmonary Vein), CL (Cycle Length). | | | | |

| Table S10: Redo procedures and PV reconnection rates according to recurrence pattern | | | |
| --- | --- | --- | --- |
| Characteristic | **Total**  N = 219 | **AF recurrence**  N = 27 | **AT/AFL recurrence**  N = 45 |
| Redo procedure | 27/219 (12.3%) | 1/27 (3.7%) | 26/45 (57.8%) |
| PVs reconnection at Redo | 9/27 (33.3%) | 1/1 (100.0%) | 8/26 (30.8%) |
| Continuous variables are shown as Mean ± Standard Deviation (SD) or Median and (Inter Quartile Range) (IQR). Discrete variables are presented as numbers and percentages (%).  Abbreviation List: AF (Atrial Fibrillation), AFL (Atrial Flutter), AT (Atrial Tachycardia). | | | |

**Figure S1**. Distribution of shortest PV-FARS_10_ by clinical AF pattern.

The distribution of shortest PV-FARS_10_ differed between paroxysmal and persistent AF. The distribution was positively skewed (right-tailed) with heavier tails in paroxysmal AF (skewness 1.60; kurtosis 3.72) compared with persistent AF (skewness 0.78; kurtosis 1.60). PV, pulmonary vein; AF, atrial Fibrillation.
