# Supplementary figures and images for: Shortest pulmonary vein atrial fibrillation cycle length identifies pulmonary vein isolation responders beyond clinical atrial fibrillation pattern: the FARS-AF II study
Source: Europace. 2026 Feb 23;28(2):euag033. doi: 10.1093/europace/euag033 (PMC12964357; doi:10.1093/europace/euag033)

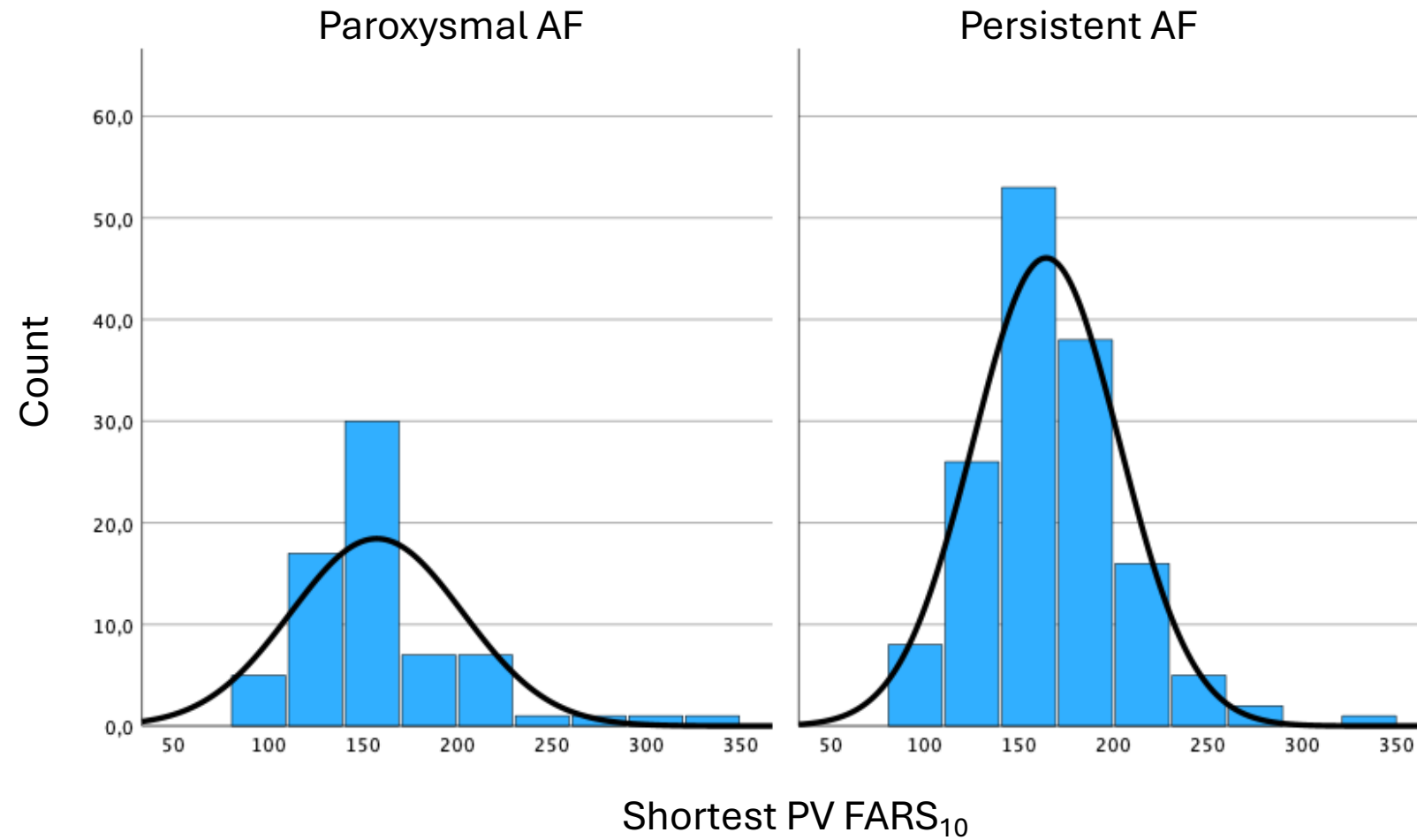

Supplement: euag033_Supplementary_Data [file euag033_supplementary_data.zip › FigureS1.pdf]
